# Success of pulpotomy with MTA in primary teeth: A systematic review and meta-analysis

**DOI:** 10.6026/973206300212574

**Published:** 2025-08-31

**Authors:** Tejal Laxman Beldar, Ashwin Muralidhar Jawdekar, Laresh Naresh Mistry

**Affiliations:** 1 Department of Pediatric Dentistry and Preventive Dentistry, Bharati Vidyapeeth (Deemed to be) University Dental College and Hospital, Navi Mumbai, India

**Keywords:** Pulpotomy, mineral trioxide aggregate/ MTA, pulp therapy, formocresol, irreversible pulpitis, primary teeth

## Abstract

The challenge of effectively managing primary teeth with irreversible pulpitis in pediatric dentistry is of interest. The clinical and radiographic success of different pulpotomy materials, including Mineral Trioxide Aggregate (MTA), in comparison to conventional materials like formocresol, ferric sulfate, calcium hydroxide, and Biodentine is reported. Therefore, it is of interest to evaluate the effectiveness of materials like MTA in pulpotomies for primary teeth with irreversible pulpitis. The meta-analysis revealed that MTA pulpotomies had a clinical success rate of 97.02% and a radiographic success rate of 94.21%, outperforming ferric sulfate, Biodentine and calcium hydroxide. CEM and Calcium Silicate Cements showed comparable success rates to MTA. Thus, MTA demonstrated superior clinical and radiographic outcomes for pulpotomy in primary teeth with irreversible pulpitis, showing statistically significant differences compared to other materials.

## Background:

The conservative management of primary teeth with irreversible pulpitis presents a significant clinical challenge in pediatric dentistry [1]. Preserving the primary dentition is crucial for maintaining function, aesthetics, and proper alignment of the permanent teeth [2]. Pulpotomy, a widely accepted procedure for treating cariously exposed primary molars, is a critical dental procedure frequently employed to manage extensively decayed primary teeth and maintain their functionality until natural exfoliation [3]. The technique involves removing the coronal part of the dental pulp, followed by placing a medicament that preserves the vitality of the remaining radicular pulp [4]. For decades, numerous materials have been used in pulpotomy, each designed to achieve optimal clinical and radiographic outcomes [5]. Mineral trioxide aggregate (MTA) has emerged as a prominent material due to its excellent biocompatibility, practical sealing ability, and regenerative properties [6]. However, the quest for the ideal pulpotomy material is ongoing, with numerous studies comparing MTA to other traditional and contemporary materials [7]. Therefore, it is of interest to report the clinical and radiographic outcomes of pulpotomy in primary teeth using various materials compared to MTA.

## Review:

This systematic review and meta-analysis investigates the success of MTA (calcium silicate-based cements) compared to other materials in pulpotomies of primary teeth with irreversible pulpitis. Using the PICOS framework, the Population (P) included primary teeth with irreversible pulpitis, the Intervention (I) involved calcium silicate-based cements (*e.g.*, MTA, CEM, Biodentine), and the Control (C) group used conventional materials (formocresol, ferric sulfate, calcium hydroxide). The Outcome (O) was clinical and radiographic success rates, and the Study design (S) focused on randomized controlled trials (RCTs). A thorough literature search was performed between March 1 and May 31, 2024, using PubMed, the Cochrane Library, Google Scholar, and Semantic Scholar. Only RCTs published in English or translated into English with full texts were included. Studies with a minimum 12-month follow-up were selected. The review adhered to PRISMA 2020 guidelines, and the detailed study selection process is shown in Table 1 (see PDF). The sources and methodology of this systematic review and meta-analysis were designed to ensure accuracy and reliability. A comprehensive literature search was conducted by two researchers between March 1, 2024, and May 31, 2024, targeting English-language studies with full texts available. The primary databases searched were PubMed and the Cochrane Library, extended to Google Scholar and Semantic Scholar for comprehensive coverage. No date restrictions were applied, and only randomized controlled trials (RCTs) were included. The review followed PRISMA 2020 guidelines, ensuring a standardized approach. Eligibility criteria included RCTs involving children under 10 years with irreversible pulpitis in primary teeth and a minimum 12-month follow-up for clinical and radiographic success. The PRISMA 2020 flow diagram of the study selection process is shown in Figure 1 (see PDF).

The risk of bias (ROB) in the studies was assessed using the Cochrane Risk of Bias II tool, with independent assessments by both researchers and resolution of discrepancies through discussion or a third reviewer. The study is registered on PROSPERO (CRD42023468690, October 2023). Meta-analysis was conducted using forest plots to evaluate pooled clinical and radiographic success rates of pulpotomy materials. Heterogeneity was assessed with the I^2^ statistic, and publication bias was examined through funnel plot analysis. Statistical analysis was performed with MedCalc software, ensuring a rigorous, unbiased comparison of MTA and other materials in primary teeth with irreversible pulpitis. This study follows PRISMA guidelines to provide reliable insights for pediatric dental practitioners. The clinical and radiographic successes of pulpotomy using MTA and other materials in primary teeth with irreversible pulpitis for 30 studies (over 2500 participants) are reported in Table 2 (see PDF). Comparison of clinical and radiographic success of pulpotomy of calcium hydroxide, Biodentine, formocresol, ferric sulfate with MTA in primary teeth with irreversible pulpitis is reported in Table 3 (see PDF). The Risk of Bias summary was assessed using the Cochrane Risk of Bias II tool (Figure 2 - see PDF). The corresponding funnel plots for detecting publication bias are available in the additional information. Clinical and radiographic success of pulpotomy using MTA in primary teeth with irreversible pulpitis for 30 studies (over 2500 participants), the forest plot and funnel plot analyses are reported in Figure 3 (see PDF) to Figure 6 (see PDF).

## Discussion:

Irreversible pulpitis is when the dental pulp becomes inflamed and damaged to the point where it cannot heal independently. This condition is usually a result of deep decay, trauma, or repeated dental procedures that irritate the pulp [35]. Key characteristics of irreversible pulpitis include severe, intense, lingering pain, especially in response to hot or cold stimuli. The pain may also be spontaneous, without any external trigger. Severe inflammation leads to irreversible damage. As the condition progresses, the pulp may become necrotic, potentially leading to infection and an abscess at the root tip. Irreversible pulpitis is believed to require more invasive treatments than pulpotomy because the pulp cannot recover independently [36]. The standard therapy for irreversible pulpitis is to remove the inflamed and damaged pulp to prevent further complications. In primary teeth, this is usually done through pulpotomy (removal of the pulp from the coronal portion) or pulpectomy (removal of the entire pulp), followed by filling the space with a suitable material [11]. If left untreated, irreversible pulpitis can lead to more severe dental issues, including abscess formation, bone loss around the tooth and potentially needing tooth extraction. The emergence of calcium silicate-based materials, particularly MTA and newer materials like Biodentine, has significantly transformed the practice of pulpotomy, especially in pediatric dentistry [37]. These materials have introduced a paradigm shift in the management of dental pulp therapy, primarily due to their superior biological properties (includes the inductive ability leading to dentin formation), clinical efficacy and long-term success rates [13]. These materials have revolutionized pulpotomy procedures by offering more biocompatible, effective and durable solutions for managing irreversible pulpitis in primary teeth. These materials have set a new standard in dental pulp therapy, leading to better patient outcomes and transforming the approach to pediatric dental care. Pulpotomy was used initially as a devitalisation procedure for inflamed pulp just for the pain to subside which is an obsolete concept now. We now prefer preservation/ regeneration approach to the earlier mummification/ devitalization practice [38]. Devitalization, preservation and regeneration reflect the evolution of the procedure from a focus on simply managing symptoms to promoting long-term dental health and natural healing. These approaches offer more sustainable outcomes, especially in pediatric patients, by maintaining the function and health of the affected tooth until it can naturally exfoliate or continue to develop (as in the case of permanent teeth) . Although several randomised controlled trials have been available reporting success of these materials, some of these with recent evidence are available with sufficient follow-up. Pulpotomy treatment failures resulting in inflammation could be noticed over a period of 1 year and beyond; hence, our study assessed the success of pulpotomy with an inclusion criterion of minimum 1-year follow-up while assessing both individual and comparative performance of various materials. We found that calcium enriched mixture, calcium silicate, MTA and Biodentine cements to have the best clinical success followed by formocresol and ferric sulfate and was lowest for calcium hydroxide. Radiographically, a similar trend was observed. In general, both the clinical and radiographic success of these materials is comparable to that of reported studies for pulp therapies of primary teeth without irreversible pulpitis. Junior *et al.* [38] reported that the success rate of MTA was higher than that of formocresol, with a statistically significant difference. Formocresol pulpotomy success was not statistically different from ferric sulphate or electrosurgery. Tewari *et al.* [37] reported that pulpotomy medicaments, except calcium hydroxide, showed success rates of more than 80%, whereas most comparisons revealed no differences. MTA, however, was found to be better than calcium hydroxide and formocresol. In comparison to MTA, calcium hydroxide, formocresol and ferric sulfate pulpotomies showed lower clinical and radiographic success. Biodentine exhibited superior clinical success however; radiographically the success was not significantly different. Junior *et al.* [38] reported that overall clinical and radiographic success rates Biodentine vs. MTA did not differ statistically in the 6-month follow-up. Coll *et al.* [39] reported that two calcium silicate cement pulpotomies success using mineral trioxide aggregate (MTA) and Biodentine were 94 percent and 90 percent, respectively. The current SRMA has a few limitations such as inclusion of fewer studies of direct comparison, Unavailability of trials with longer follow-up i.e. more than 2-3 years, variations in the identification of different calcium silicate materials. Despite such limitations, this study confirms the possibility of success of pulpotomy in primary teeth with irreversible pulpitis.

## Conclusion:

Calcium silicate-based materials are superior to formocresol and ferric sulfate. Amongst calcium silicate-based materials, CEM and calcium silicate cement shows best outcomes followed by MTA and Biodentine. Hence, we conclude our findings; pulpotomy has potential for success over 90% in primary teeth with irreversible pulpitis using calcium silicate-based materials.
